# Application of a high-throughput swarm-based deep neural network Algorithm reveals SPAG5 downregulation as a potential therapeutic target in adult AML

**DOI:** 10.1007/s10142-024-01514-9

**Published:** 2025-01-06

**Authors:** Chinyere I Ajonu, Robert I Grundy, Graham R Ball, Dimitrios Zafeiris

**Affiliations:** 1 John van Geest Cancer Research Centre, School of Science and Technology, Nottingham Trent University, Nottingham, United Kingdom; 2 Intelligent OMICS Limited, Nottingham, United Kingdom; 3 Medical Technology Research Centre, Anglia Ruskin University, Chelmsford, United Kingdom

**Keywords:** Acute myelocytic leukaemia, SPAG5, Swarm-based deep neural network, TP53 pathway, Oncogenic drivers, Therapeutic target

## Abstract

Gene‒gene interactions play pivotal roles in disease pathogenesis and are fundamental in the development of targeted therapeutics, particularly through the elucidation of oncogenic gene drivers in cancer. The systematic analysis of pathways and gene interactions is critical in the drug discovery process for various cancer subtypes. SPAG5, known for its role in spindle formation during cell division, has been identified as an oncogene in several cancers, although its specific impact on AML remains underexplored. This study leverages a high-throughput swarm-based deep neural network (SDNN) and transcriptomic data—an approach that enhances predictive accuracy and robustness through collective intelligence—to augment, model, and enhance the understanding of the TP53 pathway in AML cohorts. Our integrative systems biology approach identified SPAG5 as a uniquely downregulated driver in adult AML, underscoring its potential as a novel therapeutic target. The interaction of SPAG5 with key hub genes such as MDM2 and CDK1 not only reinforces its role in tumour suppression through negative regulation but also highlights its potential in moderating the phenotypic and genomic alterations associated with AML progression. This study of the role and interaction dynamics of SPAG5 sets the stage for future research aimed at developing targeted and personalized treatment approaches for AML, utilizing the capabilities of genetic interventions.

**Supplementary Information:**

The online version contains supplementary material available at 10.1007/s10142-024-01514-9.

## Introduction

Acute myeloid leukaemia (AML) is a white blood cell malignancy characterized by the clonal proliferation of abnormally differentiated blasts of the myeloid lineage, the accumulation of immature progenitors (blasts) in the bone marrow (BM) and blood, and the disruption of normal hemopoiesis (Hartmut Döhner and Weisdorf, Clara 2015). Managing AML is especially challenging, as only 35–40% of individuals under the age of 60 and 5–15% of patients over 60 show complete remission from the disease, emphasizing the importance of expert knowledge to provide optimal treatments to achieve the best possible outcome (Coombs et al. 2016). In recent years, advancements in the understanding of the pathophysiology of AML have led to improvements in the treatment and therapy of the disease; however, with the development and application of new, effective medications and drug combinations for patients, many new challenges and questions have arisen (Short et al. 2020). Resistance to new therapies, both primary and secondary, is still a problem, and the processes by which resistance to many of these new drugs develops are still being explored. In many patients, the complex and dynamic clonal architecture of AML remains a primary cause of response variability and secondary resistance development. Therefore, research into new molecularly targeted therapies continues to be a top goal for both younger and older patients with AML (Short et al. 2020; Thol and Ganser 2010).

Deregulated signal transduction is an attractive target for the personalized treatment of malignant disorders, and current pharmaceutical efforts have led to a considerable number of agents designed to block the catalytic activity of signalling pathway components (Scholl et al. 2008). In most cases, aberrant signal transduction may be attributed to constitutive activation of a small number of pathways, confirming the theory that, despite genetic variability, AML is caused by the dysregulation of a small number of common effector pathways. If this theory is correct, it opens up therapeutic possibilities beyond the development of genotype-specific therapies, because the best approach to treating genotypically diverse leukaemia would be to target the pathways that drive leukemogenesis and are common to transformation in a large proportion of patients (Scholl et al. 2008; Rouhi and Fröhling 2013). One of the most frequently altered genes in cancer is the tumour suppressor gene TP53. With a history that stretches back to 1979 when TP53 was discovered, the TP53 pathway has received much attention in cancer biology and oncology with the pathway being a multifaceted, complex cellular stress response pathway with downstream effects that are pertinent to its function as a tumour suppressor route. Despite advances in understanding the biology and signalling of the TP53 pathway, the TP53 family, transcriptional readouts, and effects of a variety of mutations, the pathway is still difficult to translate into the clinical setting (Borrero and El-Deiry 2021; Barbosa et al. 2019).

Sperm-associated antigen 5 (SPAG5), also known as hMAP126 and Astrin, is a microtubule-associated protein that was first discovered to be localized in the kinetochores of congressed chromosomes and closely linked with spindles throughout mitosis (Chang et al. 2001; Gruber et al. 2002). During the mitotic phase in healthy cells, SPAG5 ensures that two sister chromatids, kinetochores and microtubules are properly attached, ensuring the proper division of the sister chromatids into daughter cells (Conti 2019). New evidence on the function of SPAG5 in numerous signalling pathways has shown that it is linked to cancer development, progression, and unfavourable clinical conditions, with elevated SPAG5 expression correlated with adverse clinicopathological features and poor clinical outcomes in multiple cancer types (Wang et al. 2021; Yang et al. 2018; Zeng et al. 2022). Additionally, in 2020, Ji et al. demonstrated the potential of targeting SPAG5 for cancer treatment and concluded that an innovative approach to cancer treatment, particularly in reversing chemoresistance, could include SPAG5 antagonism (He et al. 2020). However, the clinical significance of SPAG5 in AML and its function in this disease are unclear.

To explore the role of SPAG5 antagonism as a possible treatment strategy in AML, its role in AML pathogenesis must first be established; thus, to search for additional potential therapeutic targets and gain insights into the role of SPAG5 in AML progression, a high-throughput Swarm-based deep neural network methodology was applied to evaluate SPAG5 as a potential therapeutic target as a regulator of the TP53 pathway in AML. TP53 is one of the most frequently mutated genes in AML and is associated with a more aggressive form of the disease, treatment resistance, increased risk of relapse, and poor prognosis; hence, studying and enriching this pathway may help identify biomarkers that can be used as potential therapeutic targets.

## Methodology

### Data source, extraction, and preparation

The transcriptomic profiles of paediatric and adult AML patients were collected from publicly available omics datasets accessed via the National Cancer Institute Data Portal (https://portal.gdc.cancer.gov/). The TARGET dataset (Bolouri et al. 2018)was used for the paediatric AML cohort, whereas the TCGA (Ley et al. 2013)and BEAT1.0 (Tyner et al. 2018) datasets were used for the adult AML cohort. Table 1 below shows the number of cohorts with the corresponding average age at diagnosis for each dataset. Although high-throughput data structures and gene expression signatures can be challenging to analyse owing to features such as being nonlinear and multivariate and having large structures, they do have some distinct advantages. Transcriptomic expression datasets are reproducible, robust, and tailored for broad coverage (Claudia Manzoni, Demis et al. 2016). Additionally, the extensive availability of transcriptomic data across diverse cancer types allows for robust cross-validation and comprehensive integration of findings. This abundance facilitates consistent analysis pipelines and enhances the reliability of the results. While proteomics and other omics types are valuable and could complement this work, they currently lack the same level of coverage and accessibility across datasets, which limits their integration in studies of this scale, making them excellent candidates for analysis (Kopec et al. 2005).

**Table 1:** Parameters/Selection criteria for each dataset

Dataset	Number of cohorts	Average Age at Diagnosis
*TARGET AML*	*187*	*9*
*TCGA AML*	*151*	*55*
*BEAT AML 1.0*	*228*	*53*

The elements of the TP53 pathway, as defined by the KEGG database, were identified and extracted from each dataset. This included the expression profiles of the TP53 pathway genes. These expression profiles were then used to create three distinct datasets corresponding to the BEAT, TCGA, and TARGET AML sources. The TP53 pathway was specifically targeted in this study because of its well-established role in regulating apoptosis, DNA repair, and cell cycle progression—functions frequently disrupted in AML and with strong therapeutic relevance (Granowicz and Jonas 2022). Additionally, alterations in the TP53 pathway are correlated with adverse prognoses in adult AML patients, underscoring its potential as a therapeutic target (Prokocimer et al. 2017; Zhu et al. 2023; Vadakekolathu et al. 2020).

To ensure comparability and consistency across datasets, min–max normalization was applied to scale the expression values of each dataset to a range between 0 and 1. This normalization process reduced variability arising from differences in data collection platforms and preprocessing pipelines, ensuring that all three datasets were suitable for subsequent analysis and were used as predictors in a stepwise artificial neural network to identify genes closely interacting with the TP53 pathway. To provide a comprehensive view, expression trends across the datasets were visualized in a heatmap, as shown in Supplementary File 1, which illustrates the mean comparative expression patterns across datasets. The expression trend shows that, while TCGA and BEAT share more similarities in TP53 pathway gene expression (reflecting consistency as expected within adult AML), TARGET exhibits a distinct expression pattern expected since it is a paediatric dataset, and the two conditions are clinically distinct (Farrar et al. 2016). Additionally, the expression trend serves to assess technical consistency and bias across datasets. Despite differences in patient demographics, sampling methods, and data processing protocols, the observed expression patterns remain broadly comparable, indicating that the trends are robust and not significantly influenced by technical artifacts. This provides reassurance that the findings are not only biologically meaningful but also reliable across independent cohorts.

### Stepwise neural network

To augment the TP53 pathway, as is currently understood, a stepwise artificial neural network (ANN) approach was applied, which allowed us to generate an enriched set of genes identified as expansions to the pathway. This neural network methodology allows the expansion of gene sets by identifying additional genes that interact closely and contribute to pathways. This approach has been previously proven successful in discovering and identifying novel drivers and biomarkers that can be used as therapeutic targets (Lancashire et al. 2009; Zafeiris et al. 2018). The stepwise ANN method utilized for the datasets used in this study was first described by Lancashire et al. (2008) and has been demonstrated to be effective in identifying patterns within the data by determining the optimum inputs for categorizing a specific task on the basis of the predictive performance of each variable, in this case, the expression levels of each gene in the TP53 pathway. This approach is especially helpful for biological datasets because it allows us to determine the genes most likely to account for a dataset’s variance (Zafeiris et al. 2018; Dhondalay 2013).

Neural networks present distinct advantages over traditional network enrichment analysis by leveraging their ability to uncover complex, nonlinear relationships in gene networks that are difficult for traditional methods to detect. Traditional network enrichment analysis, such as the approach described by Alexeyenko et al., enhances the statistical overlap of gene sets by incorporating network topology, making it more powerful than conventional gene set enrichment analysis (Alexeyenko et al. 2012). However, neural networks extend these capabilities by dynamically modelling interactions and capturing intricate patterns in multidimensional datasets. Unlike traditional methods that rely on predefined functional groups and statistical overlap, neural networks adaptively learn from data to reveal novel connections and biological insights, even for genes not well annotated in existing databases. This flexibility allows neural networks to provide richer, more accurate characterizations of gene relationships by integrating data from diverse omics sources, such as gene expression and protein interaction networks, without being constrained by fixed pathways or modules. Furthermore, the ability of neural networks to efficiently process high-dimensional data overcomes the curse of dimensionality, enabling the discovery of hidden interactions with enhanced scalability and robustness. This makes neural networks a transformative tool for functional genomics, offering deeper biological insights than traditional network enrichment approaches do (Lancashire et al. 2008; Tong et al. 2014).

The stepwise ANN is composed of a multilayer perceptron (MLP) with a single hidden layer containing two hidden nodes and a backpropagation algorithm that adjusts the network weight by feeding the error back through the model. A Monte Carlo cross-validation (MCCV) approach was used to stratify the data between training, validation, and testing with a 60:20:20 split, with 50 iterations used to ensure true randomization. It also utilizes an early stopping mechanism that allows the algorithm to stop early if no further improvements can be made to the model, with a minimum window threshold of 1000 iterations without improvement out of 3000 maximum iterations, which controls for possible overfitting of the data. A learning rate of 0.1 and a momentum of 0.5 were used to control the BP algorithm, and the model’s initial weights were set between 1 and − 1. The predictive power of each gene was evaluated via the mean square at the end of the process (Dhondalay 2013). The genes with the highest predictive power identified during the stepwise process as well as the genes belonging to the TP53 pathway were further analysed via the swarm-based deep neural network analysis approach, which is an improvement over methods that have been used extensively in other studies (Zafeiris et al. 2018; Dhondalay 2013; Lemetre et al. 2010; Wagner et al. 2018; Barron et al. 2018; Zafeiris et al. 2017) to predict how a given network of elements, in this case, genes interact with each other.

### Swarm-based Deep Neural Network Analysis

**Fig. 1:**
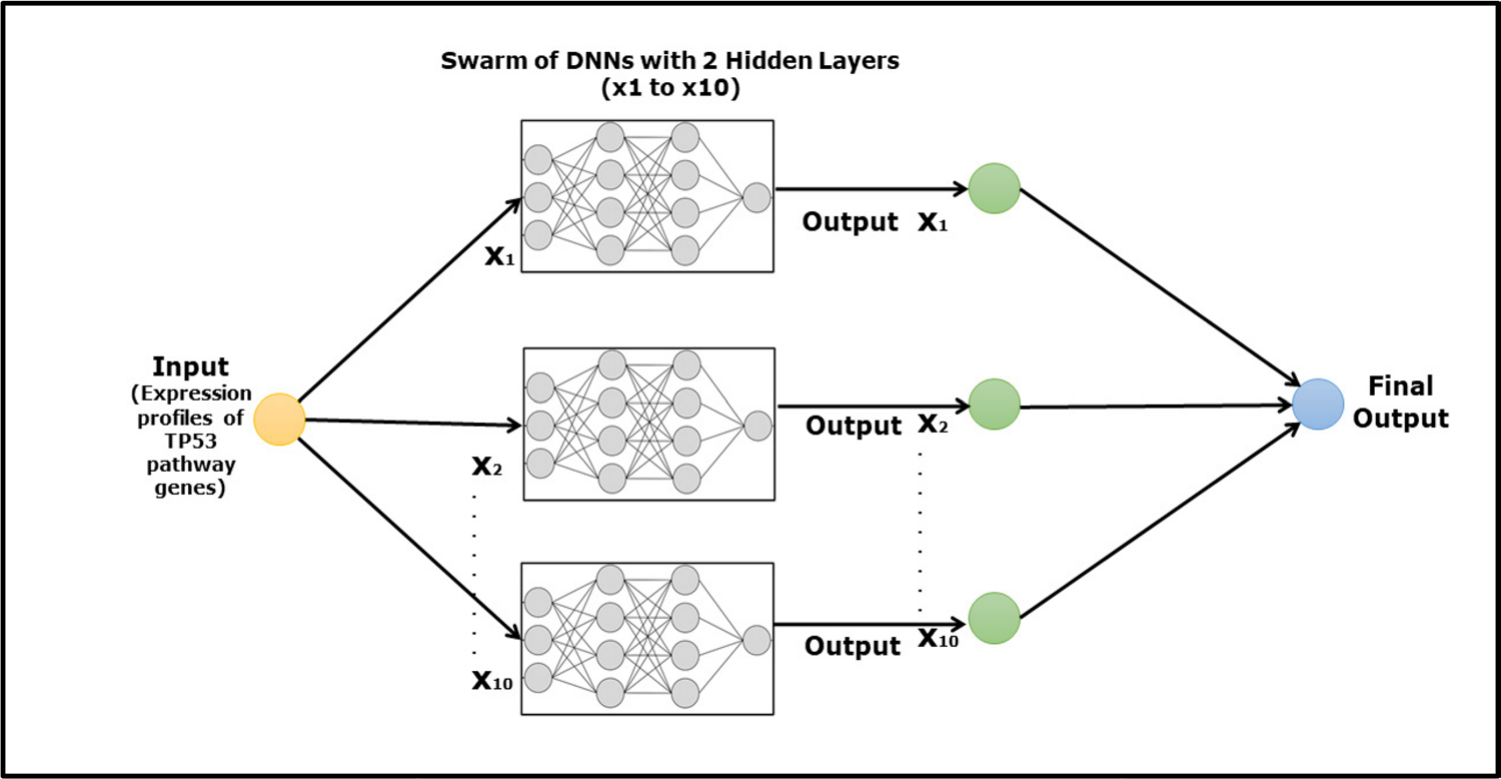
Schematic illustration of the swarm-based deep neural network used in this study. Multiple DNNs labelled X1 to X10 operate in parallel to process an input. Each DNN receives the same input and independently generates an output. These individual outputs are then aggregated to produce a final, combined output. This approach leverages the collective processing power of the swarm of ANNs to increase the robustness and accuracy of the final output. The diagram shows the input flowing into each DNN and the subsequent flow of outputs converging into a singular final output, demonstrating the collaborative computation in the swarm-based model

Artificial neural networks mimic the function of human brain neurons by transmitting signals to connected neurons. They can recognize patterns and linearly separate them by giving each input a numerical weight value and adjusting it as the data are sampled, effectively finding the best possible solution to a specific question. A Swarm-based Deep Neural Network (SDNN) algorithm was employed to model the TP53 signalling pathway, and the architecture of the SDNN consisted of a three-layer feed-forward perceptron model, which was structured with the input layer containing the total number of genes (N), a hidden layer with two units, and a single output layer (N-2-1architecture) (see Fig. 1). A back-propagation algorithm was implemented to optimize the model, facilitating the identification of the most influential genes within the system. The algorithm allows for the determination of the central role within a system of the most influential genes, and the major advantage of this methodology is that it is multifactorial and evaluates each input, allowing the magnitude of the interaction for a particular parameter to be determined, whether it is inhibitory, stimulatory, unidirectional, or bidirectional (Lancashire et al. 2009; Dhondalay 2013; Lemetre et al. 2010). The Swarm-based deep learning model was developed by combining several smaller models, and the goal of this integration was to maintain interpretability, ensuring that the model’s depth of information and machine learning capabilities were preserved.

The collaborative swarm for optimization or decision-making at each iteration offers significant benefits over traditional deep learning methods. The iterative process across multiple partitions increases adaptability and resilience, while leveraging swarm intelligence helps the model handle a wide range of scenarios, making it especially effective for dynamic and complex diseases such as cancer. This method also allows for exploring a broader solution space, potentially finding better configurations, and boosting overall model performance. Additionally, compared with traditional deep learning techniques, the collaborative swarm approach is significantly less computationally expensive; offers a high degree of biological explainability; and improves adaptability, robustness, and efficiency, making it a valuable strategy for challenging learning tasks (Zhang et al. 2021; Wu et al. 2011).

Learning was conducted over 3000 epochs with a termination factor of 1000 epochs, and the other parameters are listed in Table 2. The parameters listed in Table 2were carefully selected on the basis of prior optimization, testing, and validation performed by Tong et al. in 2014 (Tong et al. 2014), and Lemetre in 2010. These criteria were used to ensure the robustness and accuracy of the algorithm. A summary of their assessment results demonstrated high accuracy in terms of the true positive rate (TPR), correlation results, and interaction sign predictions, confirming the feasibility and reliability of these parameters. Notably, their findings revealed no significant improvement in the TPR when the number of hidden nodes increased, suggesting that the predictive ability of the algorithm is not significantly affected by this parameter. For example, this model with just two hidden nodes was found to perform better than models with more hidden nodes while requiring significantly less computational time. On the basis of these results, these parameters were implemented in the algorithm because they reflected the best performance for the time and cost of the analysis, optimizing both efficiency and effectiveness. In addition, the parameter configuration has been successfully validated and applied in multiple subsequent studies (Zafeiris et al. 2018; Lancashire et al. 2008; Tong et al. 2014; Zafeiris et al. 2017; Lemetre et al. 2009), demonstrating its robustness and reliability across diverse datasets and contexts. The optimization process ensures that the algorithm consistently delivers high predictive power under the given parameter settings, minimizing variability in the results.

**Table 2:** Interaction algorithm parameters

Parameter	Setting
*Architecture*	*N-2-1, where N = total number of genes*
*Activation function*	*Sigmoid*
*Epochs*	*300*
*Termination factor*	*1000 epochs*
*The mean square error (MSE) threshold*	*0.01*
*MSE window*	*100*
*Momentum*	*0.5*
*Learning rate*	*0.1*
*Pearson r cut-off*	*0.7*
*Number of times the whole process repeats for a single variable*	*10*

Analysing high-throughput omics data via deep learning models presents several challenges, including the high dimensionality of omics data, which can lead to overfitting and require advanced feature selection techniques. Deep learning models also lack the ability to make inductive predictions and determine the directionality of interactions (Lancashire et al. 2009). The stepwise artificial neural network approach addresses these challenges by incrementally adding layers and nodes to the network, managing complexity, and reducing overfitting risk. This method integrates feature selection within the training process, automatically identifying relevant features from omics data. Moreover, it enhances model interpretability by revealing key features for prediction. By iterative training on data subsets, the stepwise ANN approach optimizes performance with limited labelled data.

To mitigate the risk of overfitting, for both the stepwise ANN and the SDNN, independent transcriptomic datasets, specifically the TCGA, TARGET, and BEAT datasets, which are more recent, robust, and comprehensive, were employed. These datasets have provided robust validation for our findings and predictive models developed by the SDNN. Additionally, Monte Carlo cross-validation and an early stopping approach were employed. In addition, during training, the weights of the neural networks were regularized. Finally, the adopted concordance-based approach involving the use of multiple datasets, which is described in the differential driver analysis section, was a supplementary measure employed to mitigate the risk of overfitting.

### Filtering and visualizing the interaction matrix

A large matrix of interaction scores was generated by averaging the values across 10 iterations of the SDNN process until all the gene probes were used as outputs. The results were then sorted from largest to smallest with the initial values to obtain the distribution of the downregulated and upregulated interactions, followed by their absolute values to determine the strength of each interaction. The top 500 gene interactions based on the absolute interaction strength as well as interactions with SPAG5 specifically were then selected for model visualization via Cytoscape v3.10.2 (Shannon et al. 2003). Additionally, to reveal the significant module and hub genes closely interacting with SPAG5, MDM2, CDK1, and TP53, from their selection, the interactome models were analysed via MCODE (parameters: degree cut-off-4, node score cut-off-0.2, K-Core-2, and Max. depth-1000). Visualizing with Cytoscape makes it easier to create an interactive map with nodes representing sources and targets, edges representing relationships, the thickness of edges indicating the intensity of the association, different edge colours indicating whether the interaction is inhibitory or stimulatory, and arrows indicating the directionality of the pathway (Villaveces et al. 2015).

The approach further involved analysing an extensive interaction matrix generated from the network inference, which comprised 𝑛 = 83 genes. By considering each gene as an input, the model was designed to infer directed gene‒gene interactions/associations in a pairwise manner. The systematically formed associations resulted in n(*n* − 1) = 6,806 possible interactions, and this matrix enabled us to capture all potential gene‒gene relationships within the enriched TP53 pathway context. To specifically assess the interactions involving SPAG5, the interaction matrix was filtered to isolate associations with SPAG5. This filtering was applied to the interaction matrix generated from the three datasets—TCGA, TARGET, and BEAT—to ensure a robust and comparative analysis of SPAG5 interactions with key therapeutic targets. The absolute values of the interaction strengths were then employed to evaluate the robustness and potential biological relevance of each association, with higher values indicating stronger interactions.

Changes in the strength of interactions between key genes can significantly influence the stability and adaptability of the gene regulatory network. Stronger interactions may intensify regulatory control, potentially reinforcing feedback loops and stabilizing specific cellular states, such as cell cycle arrest or apoptosis, which are crucial in controlling cancer progression. Conversely, weakened interactions might reduce regulatory influence, destabilizing the network and allowing for unchecked cell proliferation or impaired apoptotic responses. These shifts could lead to network reorganization, with alternative pathways or compensatory mechanisms emerging to maintain functionality, though not always effectively. Understanding these dynamics is essential, as it enables us to predict how the network might respond to therapeutic interventions and to identify potential vulnerabilities that can be targeted to disrupt oncogenic processes (Li and Wang 2014; Rizi et al. 2021). These dynamics can be exploited by targeting key regulatory hubs with therapeutics that either weaken oncogenic interactions, destabilising cancer-promoting networks, or strengthen tumour-suppressing networks to restore normal function. Additionally, identifying compensatory mechanisms can guide combination therapies to prevent adaptive resistance and enhance treatment efficacy (Carels et al. 2023).

### Differential driver analysis

To expand the understanding of the molecular pathways underlying cancer initiation and progression, transcriptomic-based data need to define and prioritize cancer drivers (Newberg et al. 2018)but one of the greater challenges is deciding upon the selection criteria that will allow for the elucidation of a driver or therapeutic target. Therefore, by adding the sum of weights that every source gene confers on each target and vice versa, it becomes possible to rank our candidates by their overall impact on the network as drivers, defined by the overall impact they have on other genes, and as receivers, defined by the overall impact other genes have on them. The SDNN process enables iterative quantification of the influence that various genes could have on the level of gene expression until all the genes in the data have been quantified, which enables us to develop a set of low-bias criteria determined by the impact each gene has on the network, the degree of influence source genes have on targets and the degree of influence a target has imparted on by source genes (Lemetre et al. 2010). The advantage of this method is that it is not biased towards single strong interactions, as it is simply the overall impact of each gene on the network allowing for the discovery of drivers with a relatively smaller but consistent impact on the overall network, which might otherwise be hidden by the selection criteria. These genes, while not obvious drivers, are likely key components of the disease system, and analysing them can provide us with a more comprehensive and unbiased view of the disease.

The methodology used in previous studies was then expanded with differential driver analysis, a comparative analysis that allows for the assessment of each potential driver’s impact and performance across multiple distinct patient groups. For the adult cohort, which included two datasets, the mean amount of influence for each gene was calculated and compared to that of the paediatric group. Differential driver analysis enhances the identification of key regulatory genes in different AML cohorts by allowing the assessment of each potential driver’s impact and performance across multiple distinct patient groups. For example, in the adult cohort, which included two datasets, the mean amount of influence for each gene was calculated and compared to that of the paediatric group. This comparative analysis helps identify genes that may drive AML pathogenesis in specific age groups, providing insights into age-specific differences in disease mechanisms. By calculating the mean amount of influence for each gene in each age group and comparing these values, researchers can identify genes that are more influential in driving AML in one age group than in another. This analysis can reveal the age-specific molecular mechanisms underlying AML and help identify potential therapeutic targets that are specific to certain age groups.

## Results

### Augmenting the TP53 pathway

Using the stepwise approach described in Sect. 2.2, the best predictive markers for the TP53 pathway were identified. Each known pathway gene was used as a predictor in the ANN algorithm, which allowed the ranking of all genes in the transcriptome by their association with each pathway gene. This process was repeated ten times for each pathway gene, and only the genes that were consistent across all repetitions were selected to minimize false discovery. The list of concordant genes was then combined with the known elements of the TP53 pathway to create an enriched version of the TP53 pathway, which was then used for SDNN inference to determine how the novel genes interact with known pathway genes as well as each other. SPAG5 emerged as one of the novel enriched TP53 pathway genes from the stepwise analysis. Interestingly, SPAG5 was found to be one of the most concordant and consistent across all 10 analyses, implying that SPAG5 interacts with and modulates the activity of several components of the TP53 pathway.

### Interaction distribution

**Fig. 2:**
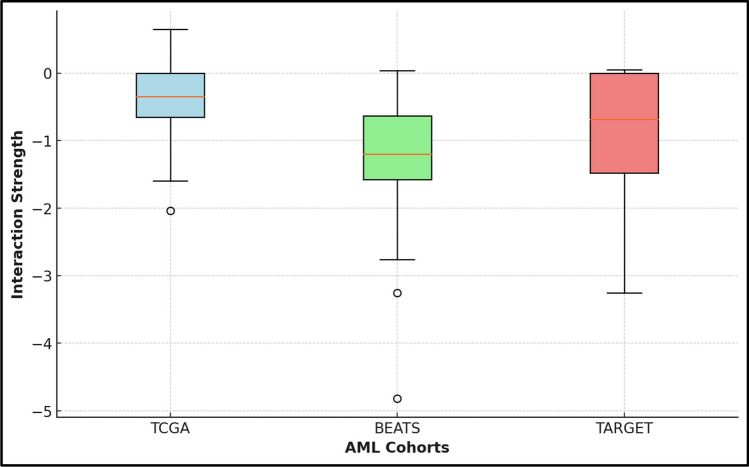
Distribution of interaction strengths sorted by interaction scores for associations involving SPAG5 in the TP53 pathway model with enriched pathway genes among all 3 cohorts. Each box plot shows the median interaction strength (indicated by the horizontal line within each box), the interquartile range (the box itself), and the overall range (the vertical lines extending from the boxes). Notably, TCGA has a median interaction strength of approximately − 0.5, with outliers near − 3. BEAT has a median closer to −0.75, with outliers at approximately − 3 and − 4. TARGET has a median of approximately − 1.5, with a more consistent spread of data and no significant outliers compared with the other two methods. The box plot indicates that the interaction strengths are predominantly negative across all three cohorts—TCGA, BEAT, and TARGET

Overall, the distribution of the average of the ten interaction values showed a greater majority of negative interactions in all cohorts, with interaction scores ranging from − 4.8 to 0.6. In the TCGA cohort, the strongest negative interaction was − 2, involving SPAG5 and MDM2, and the strongest positive interaction strength was 0.6, involving SPAG5 and CDK1, which appeared to be the strongest positive interaction among the 3 cohorts. Figure 2 shows a graphical representation of the distribution of interaction strengths from the three AML cohorts. The figure highlights the maximum and minimum interaction strengths in each case. In the TCGA interaction matrix, MDM2 exhibited the strongest interaction with SPAG5, with an absolute interaction value of 2.04. Similarly, in TARGET and BEATS, MDM2 showed a high interaction strength, with absolute values of 2.53 and 1.20, respectively, reinforcing the potential biological significance of this interaction. In the TARGET dataset, CDK1 had the fourth strongest association, with a notable absolute interaction value of 3.06, indicating a robust interaction with SPAG5. In the BEAT and TCGA datasets, CDK1 also strongly associated with SPAG5, further supporting the consistency of this interaction across datasets.

### Interaction models

**Fig. 3:**
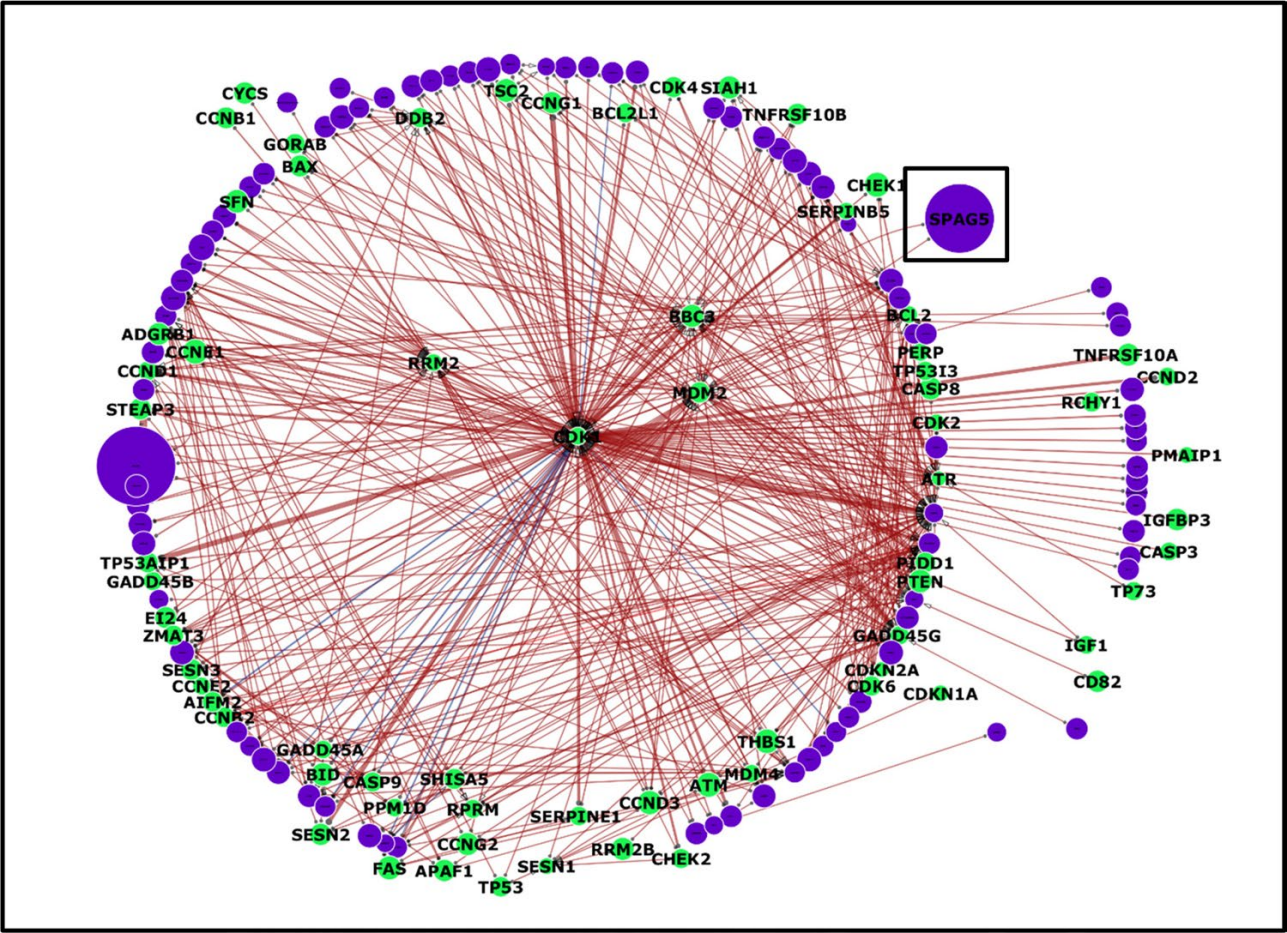
The circular layer interactome model visualizes the top 500 interactions within the TP53-enriched pathway specific to the TCGA AML dataset. Arrow directionality represents interactions from source genes, which influence target genes, which are influenced. Node size is correlated with the overall influence of a gene in the model. The green nodes represent TP53 pathway genes, whereas the purple nodes represent genes from the enriched pathway. The thickness of the edge indicates the strength or weight of the interaction, with blue edges representing positive or upregulated interactions and red edges indicating negative or downregulated interactions. The network highlights MDM2 and CDK1 as significant hubs due to their extensive interactions, emphasizing their critical roles in cellular processes and potential implications for disease mechanisms. These findings further show that SPAG5 is one of the most influential genes in the pathway. This visualization aids in comprehending how these genes interact within the network, which is crucial for exploring disease pathways and developing targeted therapeutic strategies

**Fig. 4:**
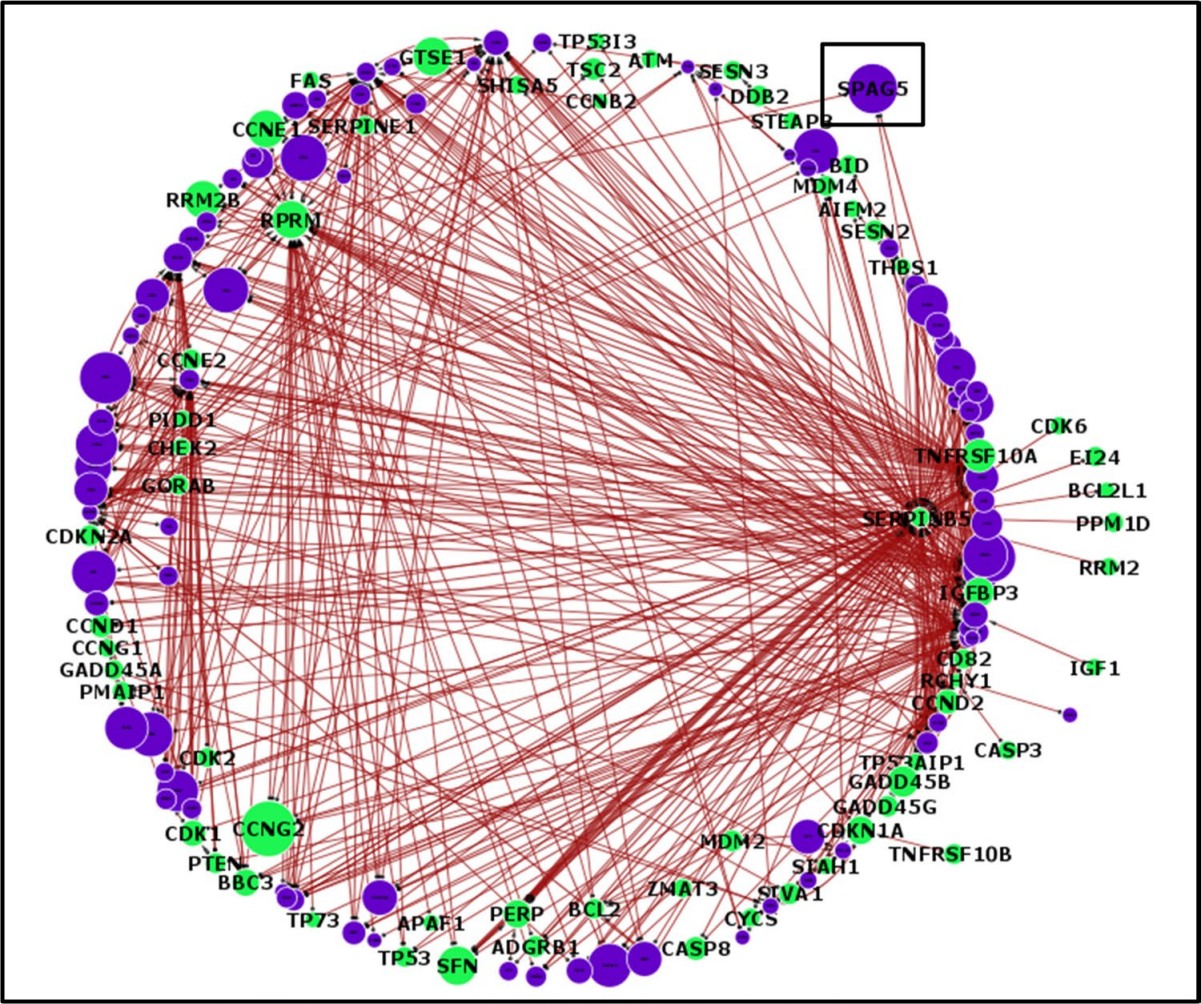
Circular layer interactome model for the top 500 interactions in the TP53-enriched pathway for the BEAT1.0 AML dataset. Directional cue: the round side of the edge indicates the source gene, and the arrows represent the target gene. The size of each node is a representation of the amount of influence each gene has on the model as a whole. The green nodes represent the TP53 pathway genes, whereas the purple nodes represent the enriched pathway genes. The thickness of the edge indicates the strength or weight of the interaction, with blue edges representing positive or upregulated interactions and red edges indicating negative or downregulated interactions. The interactome model again revealed SPAG5 as one of the most influential genes in the pathway

**Fig. 5:**
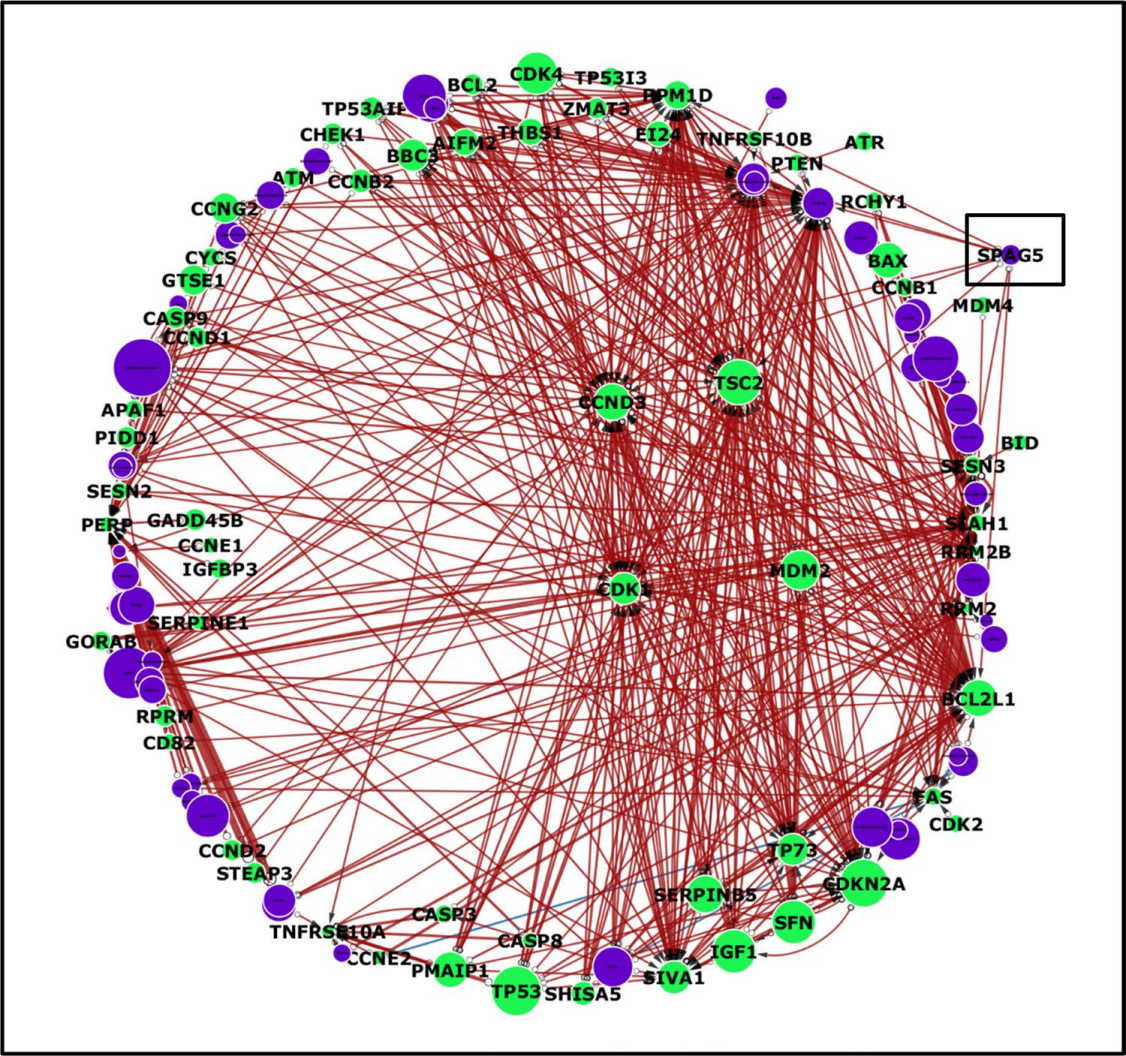
Circular layer interactome model for the top 500 interactions in the TP53-enriched pathway in the TARGET AML dataset. Directional cue: the round side of the edge indicates the source gene, and the arrows represent the target gene. The size of each node is a representation of the amount of influence each gene has on the model as a whole. The green nodes represent the TP53 pathway genes, whereas the purple nodes represent the enriched pathway genes. The thickness of the edge indicates the strength or weight of the interaction, with blue edges representing positive or upregulated interactions and red edges indicating negative or downregulated interactions. CDK1 and MDM2 are major hubs and influential genes; however, SPAG5 is one of the least influential genes

The top 500 interactions were selected by filtering them on the basis of the largest absolute value and are shown in Figs. 3, 4 and 5. Among the top 500 interactions in each cohort, all interactions were downregulated (negative) in the BEAT AML dataset, whereas the other cohorts differed slightly, with approximately 98% downregulated interactions and only a few upregulated (positive) interactions. Additionally, the models from the adult cohorts consistently revealed SPAG5 as one of the top three influential genes, as shown by the size of the SPAG5 nodes in the TCGA and BEAT1.0 cohorts’ interactomes, corresponding to the gene’s overall value in the network, determined by summing the interaction weights. However, the reverse was the case for the TARGET dataset, as SPAG5 emerged as one of the least influential genes, as shown in Fig. 5.

**Fig. 6:**
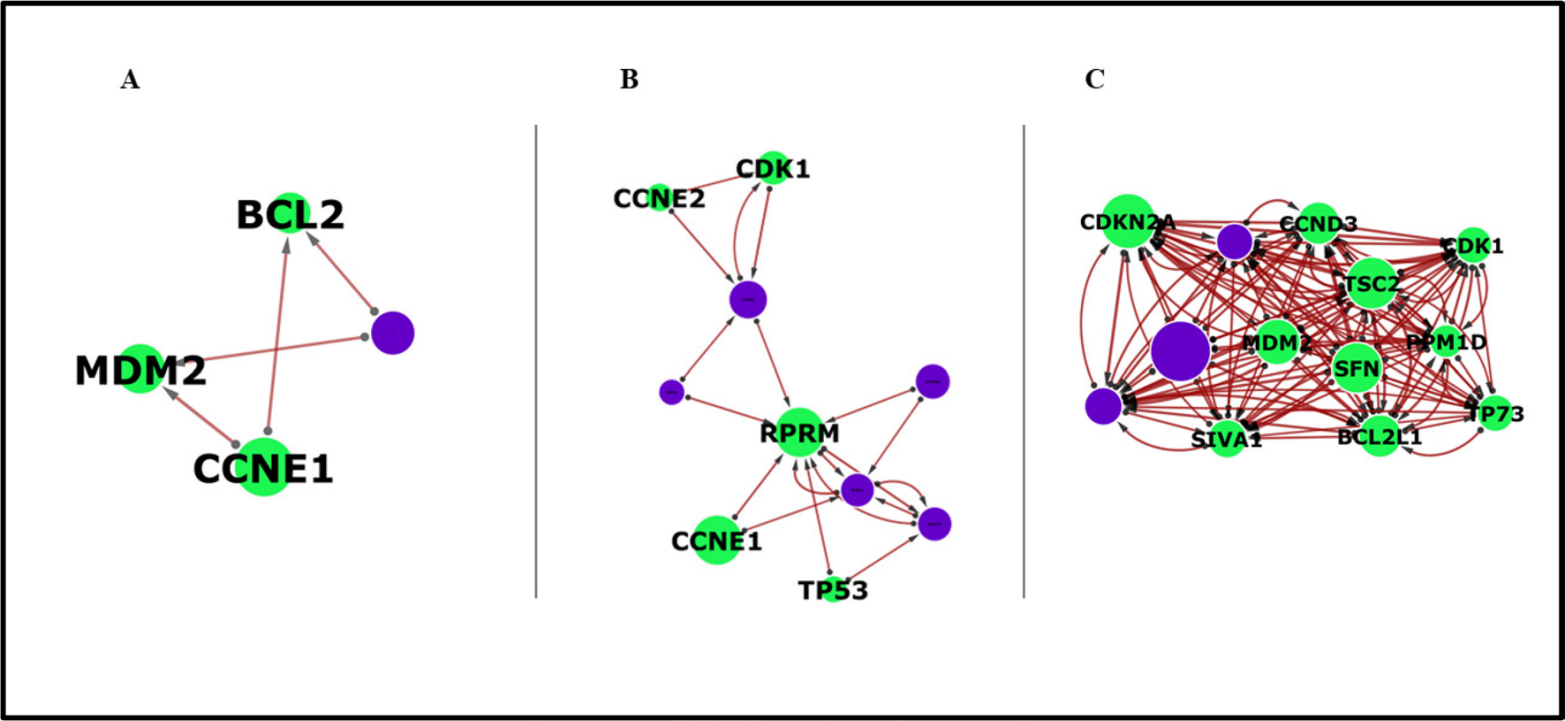
Significant module obtained from the gene‒gene interaction networks of SPAG5, MDM2, CDK1, and TP53 for the interactomes via MCODE. Arrow directionality represents interactions from source genes, which influence target genes, which are influenced. Node size is correlated with the overall influence of a gene in the model. The green nodes represent TP53 pathway genes, whereas the purple nodes represent genes from the enriched pathway. The thickness of the edge indicates the strength or weight of the interaction, with blue edges representing positive or upregulated interactions and red edges indicating negative or downregulated interactions. (A): The TCGA module revealed a tightly interconnected subnetwork involving four key nodes and four edges with four genes (BCL2, MDM2, CCNE1, AIMP2). (B): The BEAT module shows a gene‒gene interaction network centred around RPRM, highlighting its interactions with various TP53 pathway genes, including TP53, CDK1, CCNE1, and CCNE2, with ten nodes and nineteen edges. (C): The TARGET module shows a dense gene‒gene interaction network centred around MDM2, with significant connections to key regulatory proteins such as CDKN2A, CDK1, CCND3, TSC2, and TP73

For the MCODE analysis, BCL2, MDM2, and CCNE1 were identified as the TP53 pathway hub genes in the gene‒gene network analysis for TCGA. CDK1, CCNE1. CCNE2, TP53, and RPRM were the TP53 pathway hub genes for BEAT, whereas BCL2L1, CDKN2A, MDM2, CCND3, TSC2, CDK1, TP73, SIVA1, SFN, and PPM1D were the TP53 pathway hub genes for TARGET (see Fig. 6).

### Differential driver analysis

**Fig. 7:**
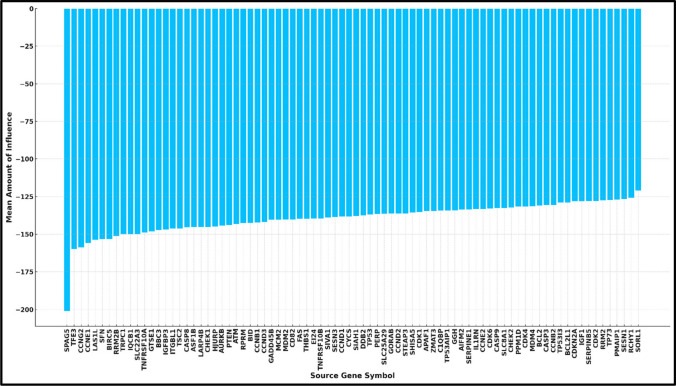
Mean amount of influence of source genes on adult AML pathways across the TCGA and BEAT1.0 AML datasets. This bar chart illustrates the average influence of each source gene within the augmented TP53 pathway for adult AML patients, with all values being negative, indicating a downregulating effect. Each bar signifies a gene prominently featuring SPAG5 as the most influential gene, significantly surpassing others and underscoring its pivotal role in the pathways associated with adult AML. The bars are arranged in descending order, visually demonstrating the decreasing influence of each gene and elucidating their relative importance in the disease context. The x-axis displays the source gene symbols, and the y-axis measures their influence, making this chart a vital resource for pinpointing potential therapeutic targets and deciphering the molecular mechanisms involved in adult AML

One significant disadvantage of traditional interaction maps is that, for clarity, a small number of the strongest interactions are showcased. However, this tends to hide the impact of genes with individually weaker but more consistent interactions that apply pressure to the entire network. To determine whether any genes with such characteristics were present and to verify the relevance of the strongest interactors, a driver analysis was performed on the results by summing all the weights leading from and to each individual gene (Zafeiris et al. 2018) In accordance with the interactomes shown, SPAG5 was identified as the most influential gene on the basis of the weight of each parental gene in the TCGA and BEAT1.0 patient cohorts. On the other hand, this was not apparent in the paediatric group, as SPAG5 emerged as the forty-second most influential gene from the TARGET cohorts. For the adult cohort, which included the TCGA and BEAT cohorts, the mean amount of influence was determined and visualized in a bar chart, as shown in Fig. 7.

## Discussion

Gene interactions within specific pathways offer crucial insights into the mechanisms that drive cancer progression, thereby aiding in the development of precise treatment strategies (Liu et al. 2020). Genes that interact closely within specific pathways identified through transcriptomic investigations can be better understood mechanistically through pathway enrichment analysis (Chicco and Agapito 2022; Reimand et al. 2019). Consequently, via a high-throughput neural network analysis, this study aimed to augment the TP53 pathway and explore the potential of the pathway genes as therapeutic targets for AML by first establishing their role in the pathogenesis of AML. Pathway enrichment analysis revealed a series of novel genes that closely interact with the TP53 pathway in AML, including SPAG5. Additionally, the interaction model and driver analysis highlighted the role of SPAG5 as a key downregulating driver in adult AML, with MDM2 and CDK1 emerging as major hubs across different patient groups. This has facilitated a deeper, unbiased examination of the variances in gene drivers of AML via the TP53 pathway, which could be attributed to imbalances caused by the disease, and these findings are discussed further below.

### Role of the TP53 pathway in AML progression

The TP53 pathway is hypothesized to play a crucial role in inhibiting the phenotypic and genomic alterations that are often associated with cancer development through a complex interplay of vital cellular processes such as cell division, maintenance of genomic stability, apoptosis, autophagy, the immune response, and regulation of the tumour microenvironment (Boutelle and Attardi 2021; Marei et al. 2021). These processes are crucial for suppressing the oncogenic potential that can lead to malignancy. The preponderance of negative interactions (downregulation) observed in the distribution of interaction strengths, interactomes, and overall influence of the TP53 pathway genes (Figs. 2, 3, 4, 5 and 6) underscores the fundamental role of the pathway in tumour suppression. Negative regulation helps maintain cellular integrity by ensuring that the necessary cellular activities are downregulated when they are not needed or could be harmful if left unchecked (Lemmon et al. 2016). Moreover, tumour-suppressing pathways such as TP53 are commonly thought to function as a signal termination mechanism; therefore, the overall downregulation of genes is likely paramount to its function as a tumour-suppressing pathway, which further reinforces the downregulatory properties of SPAG5 when included in the pathway as a regulatory mechanism (Brandon et al. 2016). Such regulatory mechanisms further aid in preventing the risk of ongoing signalling induced by drivers, oncogenic mediators, or mutant genes (Osborne et al. 2004).

### SPAG5 as a key regulator and its interactions in adult AML

In healthy cells during mitosis, SPAG5 ensures the proper attachment of sister chromatids, kinetochores, and microtubules, ensuring accurate division of chromatids into daughter cells (Thein et al. 2007). In this study, SPAG5 was identified as a highly influential gene and a downregencer of adult AML via the TP53 pathway. The downregulation of SPAG5 in adult AML could disrupt the TP53 pathway, leading to impaired function of TP53 and other pathway genes. This impairment compromises the tumour-suppressing function of the pathway and reduces the ability of TP53 to activate its target genes, resulting in decreased cell cycle arrest and apoptosis with increased cell proliferation. Consequently, leukaemia cells can proliferate uncontrollably, contributing to the progression of adult AML. The loss of TP53-mediated control over cell growth and survival allows for the accumulation of genetic mutations and resistance to apoptosis, further promoting the malignancy and aggressiveness of adult AML (Shetzer et al. 2016).

The most potent negative interaction documented in the TCGA cohort study was between SPAG5 and MDM2, which exhibited an interaction intensity of −2.04. This finding underscores the significant inhibitory dynamics between these two genes within the network. SPAG5’s role in the TP53 pathway is defined by its regulation of key players, with a complex and context-dependent impact on gene interactions. While it generally has a downregulating effect, its specific interactions could vary. For example, with MDM2, which inhibits TP53, SPAG5 may further inhibit TP53 by modulating MDM2 activity. Conversely, the positive interaction of SPAG5 with CDK1 may enhance cell cycle progression, potentially contributing to uncontrolled cell proliferation during cancer development. Furthermore, MDM2 emerged as a central hub within the interactomes and was consistently identified as one of the most targeted genes, as detailed in Figs. 3 and 5. This prominence within the interactome highlights its critical role in the regulatory framework of the pathway. Previous studies have shown that inhibiting MDM2 has the potential to reduce leukemic cell growth and that antagonizing MDM2 can induce TP53-dependent apoptosis in AML cells, suggesting that this is a promising novel therapeutic strategy for AML patients (Kojima et al. 2005; Kandarpa et al. 2019; Ho et al. 2022). These insights suggest potential avenues for targeted therapies in AML that could exploit the SPAG5-MDM2 interaction to modulate the pathway favourably, enhancing the apoptotic response in AML cells and potentially improving patient outcomes.

In addition, CDK1 was identified as a critical node within the interactomes, as illustrated in Figs. 3 and 5, where it stands out as one of the most significant hubs and a frequently targeted gene. For the TCGA cohort, the strongest positive interaction, with an intensity of 0.6, was noted between SPAG5 and CDK1. This interaction highlights a significant regulatory linkage that may influence the behaviour of these genes in cancer pathways. Interestingly, phosphorylation typically regulates CDK1 activity during the cell cycle, which is critical for the proper division and differentiation of hematopoietic cells. Disruptions in this regulation are implicated in the onset and progression of leukaemia, highlighting the importance of the function of CDK1 in both normal and pathological states (Payton et al. 2006). The role of CDK1 in haematopoiesis and leukemogenesis has been increasingly recognized, particularly because the phosphorylation of CDK1 at both its N- and C-termini can have profound effects on these processes (Fried and Friedman 2005), and CDK1 plays an important role in the treatment response of AML (Hedblom et al. 2013; Biggs et al. 2006). These insights into the role of CDK1 confirm the potential for developing targeted therapies that exploit its central role in cell cycle regulation and apoptosis to combat AML. The dynamic interaction between CDK1 and other key molecules, such as SPAG5, provides valuable targets for therapeutic intervention, aiming to disrupt the proliferative capacity of leukaemia cells and promote their elimination.

Additionally, the significant modules obtained from the gene‒gene interaction network of SPAG5, MDM2, CDK1, and TP53 for the TCGA and BEAT interactomes via MCODE (see Fig. 5A and B) revealed a tightly interconnected subnetwork involving key genes (BCL2, MDM2, CCNE1, CCNE2, CDK1, and RPRM), most of which are critical for regulating apoptosis, the cell cycle, and stress responses (Reed 1997; Nag 2013; Elkholi et al. 2019). The interaction of SPAG5 with these genes implies that modulating SPAG5 in adult AML could significantly impact the functionality of this module, hence reinforcing its importance in adult AML pathogenesis and its potential as a therapeutic target in adult AML.

### Therapeutic implications of SPAG5 and interactome analysis

Potential therapeutic strategies to target SPAG5 in adult AML include the use of SPAG5 inhibitors to disrupt SPAG5 interactions, combination therapy with other targeted drugs such as CDK1 and MDM2 inhibitors, the exploration of immunotherapy approaches targeting SPAG5-expressing cells, the use of gene editing to knock down SPAG5 expression, and the screening of natural compounds that inhibit SPAG5 or its interactions. These strategies aim to restore the normal function of SPAG5, inducing cell cycle arrest and apoptosis in leukaemia cells. On the basis of the findings presented above, the challenge becomes deciding which part of the pathway to modulate and how to achieve the desired response and stop or reverse the progression of AML. To this effect, the top 500 strongest interactions were extracted for each dataset on the basis of the absolute value of their weight within the algorithm to reduce complexity and select the most likely candidates for therapy. By doing so, we were able to increase the understanding of the TP53 pathway while including novel elements determined to be important and providing a greater depth of information than was previously available. Among all the TP53 and enriched pathway genes, SPAG5 was identified as the most influential gene for the TCGA and BEAT AML patient groups on the basis of the average strength of influence across the two datasets (see Fig. 7), indicating that SPAG5 is a key driver and potential therapeutic target for AML. Additionally, inhibiting sperm-associated antigen 6 (SPAG6), another member of the sperm-associated antigen family, has been shown to suppress the growth of malignant myeloid hematologic cell lines through the modulation of caspase proteins and TP53, highlighting its potential as a therapeutic target (Yang et al. 2015).

Along with the previously noted interactions and regulatory relationships involving SPAG5 with MDM2 and CDK1, it is evident that SPAG5 plays a significant role in modulating the TP53 pathway. The specific interaction between SPAG5 and key regulatory genes such as MDM2 and CDK1 suggests that SPAG5 could influence these pathways by inhibiting their functions. Given its involvement in these pivotal interactions, SPAG5 could contribute to the prevention of genomic instability, which is a hallmark of cancer development, including AML. The ability of SPAG5 to modulate the activity of the TP53 pathway, particularly through interactions that lead to downregulation or functional inhibition of pathway components, supports its potential as a target for therapeutic strategies aimed at enhancing the tumour-suppressive capabilities of this pathway.

### Age-specific differences and future therapeutic strategies

Importantly, however, SPAG5, which is not a top driver in the TARGET cohort, implies a significant difference between the molecular landscape of the pathways and how they can be targeted in different AML age groups, which is already supported by current research (Shahzya Chaudhury, Caitríona O’Connor et al. 2018; Ma et al. 2018; Aung et al. 2021). Within the datasets used for this analysis, the mean age for the TARGET cohort was 9 years, whereas those of the TCGA and BEAT cohorts were 55 and 53 years, respectively, and the two AML age groups exhibited distinct features, as SPAG5 was found to be a key driver only in the adult cohort, providing further evidence for the specificity of SPAG5 and increasing its value as a therapeutic target. Age is a significant risk factor for cancer, but its specific influence on the molecular landscape of cancer remains unclear. Research into pathway alterations in different cancer types has begun to reveal the nuanced changes associated with age, resulting in findings that highlight differences in oncogenic pathways, indicating a crucial role for age-related factors in cancer initiation and progression (Chatsirisupachai et al. 2021). Consequently, in this study, the fact that SPAG5 was a key driver in adult AML patients but not in paediatric AML patients could be due to age-specific differences in the molecular mechanisms underlying the disease.

Finally, there are significant differentiating factors to consider regarding potential therapeutic targets, as the epigenetic landscape and leukemic niches of AML have been attributed to the age of the cell of origin, the incidence, and the type of mutations in epigenetic modulators, presenting an additional layer of complexity. Previously, the treatment for adult AML was extrapolated to paediatric AML, assuming that any therapeutic targets discovered in an older population could be applied to treating younger patients, thus presuming that the conditions of both children and adults are identical or at least close enough to share treatment options (Shahzya Chaudhury, Caitríona O’Connor et al. 2018; Ma et al. 2018; Obszański et al. 2022; Newcombe et al. 2018). This modulation of the signalling pathway has provided advanced insights into crossroads for understanding AML progression and classification and presents new avenues for therapeutic approaches in different patient groups, allowing for a larger knowledge base to inform paediatric-specific and adult-specific treatment.

## Conclusion

This study identifies SPAG5 as a critical downregulating driver in the pathogenesis of adult AML, with significant interactions involving therapeutic targets such as CDK1 and MDM2. These interactions play essential roles in regulating the cell cycle and apoptosis, positioning SPAG5 as a promising therapeutic target with the potential to increase the efficacy of existing AML treatments. These findings contribute to a deeper understanding of SPAG5’s regulatory dynamics and offer a foundation for the development of targeted genetic interventions and personalized oncology therapies.

While the results provide valuable insights, future validation work is necessary to strengthen the findings. Experimental and in vitro studies could confirm the functional roles of SPAG5 and its interactions within the TP53 pathway. Additionally, validation via AML proteomic datasets could provide critical evidence of the impact of SPAG5 on protein-level regulatory dynamics, further elucidating its influence on key processes such as apoptosis and cell cycle regulation. These efforts would refine the understanding of the mechanistic role of SPAG5 and its therapeutic potential. Future studies should also explore the relevance of SPAG5 in paediatric AML and its broader implications across other cancers.

Overall, the findings from this study represent a significant advancement in understanding SPAG5’s role in AML, offering new opportunities for therapeutic innovation. Translational research incorporating experimental validation and proteomic analyses will be pivotal in converting these computational insights into effective clinical applications.

## Electronic supplementary material

Below is the link to the electronic supplementary material.

ESM 1 (PDF 399 KB)

## Data Availability

No datasets were generated or analysed during the current study.
