# Supplementary figures and images for: Application of a high-throughput swarm-based deep neural network Algorithm reveals SPAG5 downregulation as a potential therapeutic target in adult AML
Source: Funct Integr Genomics. 2025 Jan 6;25(1):8. doi: 10.1007/s10142-024-01514-9 (PMC11703901; doi:10.1007/s10142-024-01514-9)

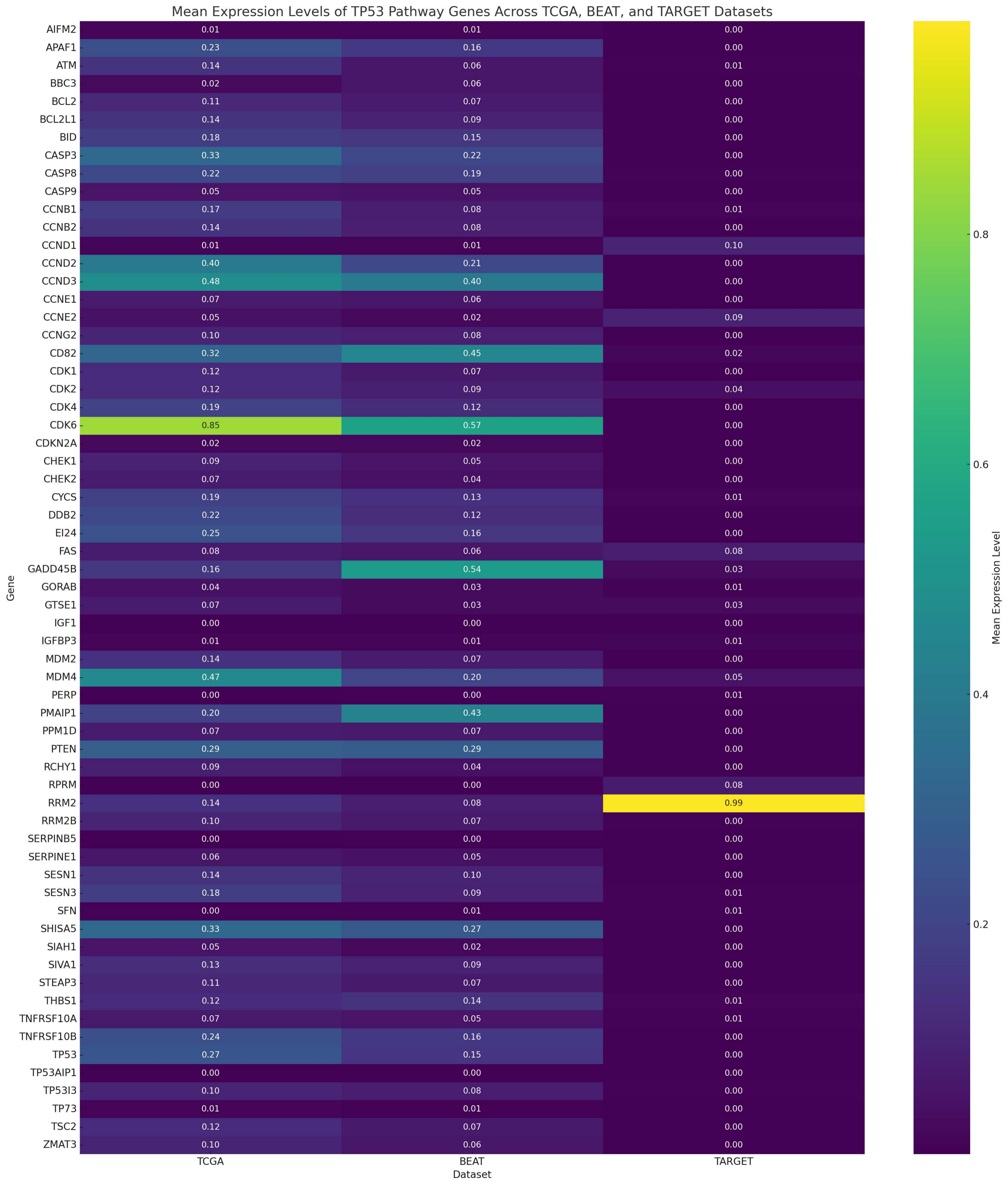

Supplement: Supplementary file 1 — (PDF 399 KB) [file 10142_2024_1514_MOESM1_ESM.pdf]
